# Supplementary figures and images for: Engineering of a high-fidelity Cas12a nuclease variant capable of allele-specific editing
Source: PLoS Biol. 2024 Jun 12;22(6):e3002680. doi: 10.1371/journal.pbio.3002680 (PMC11168656; doi:10.1371/journal.pbio.3002680)

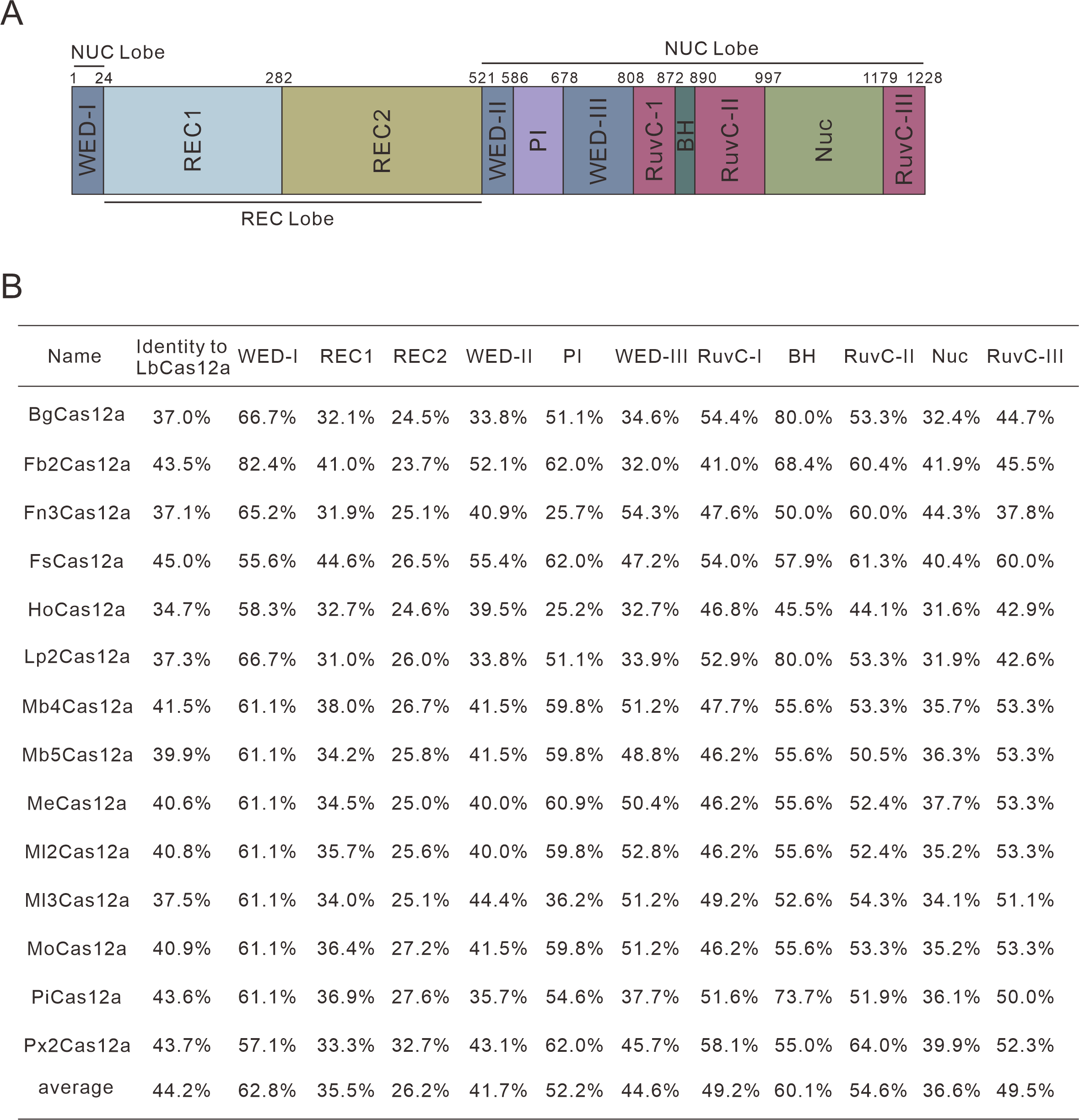

Supplement: S1 Fig — (A) Domain organization of LbCas12a [40]. (B) The percentages of protein identity between the newly identified Cas12a orthologs and LbCas12a. (TIF) [file pbio.3002680.s001.tif]

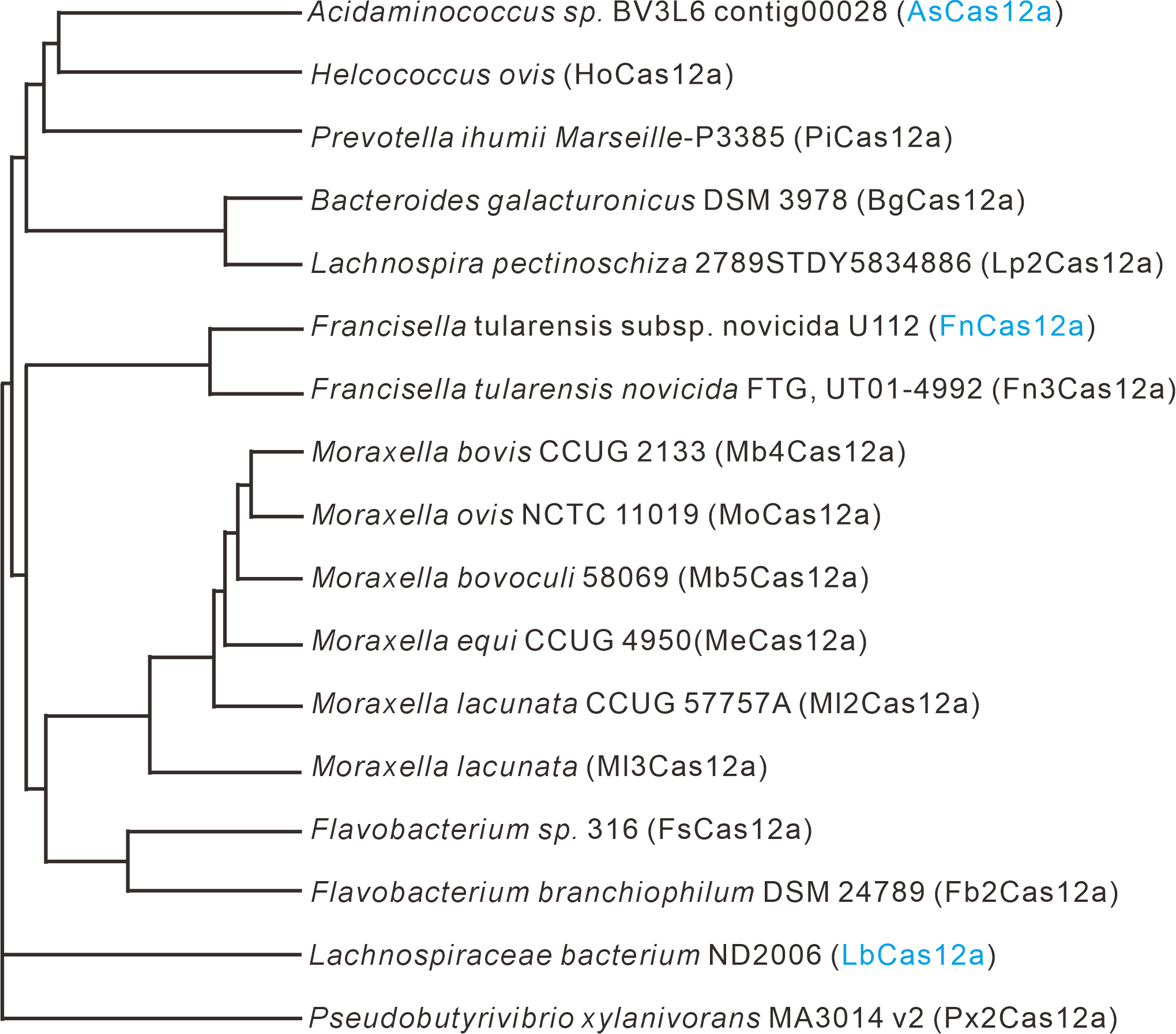

Supplement: S2 Fig — Three validated Cas12a orthologs (blue colour) are used as references. (TIF) [file pbio.3002680.s002.tif]

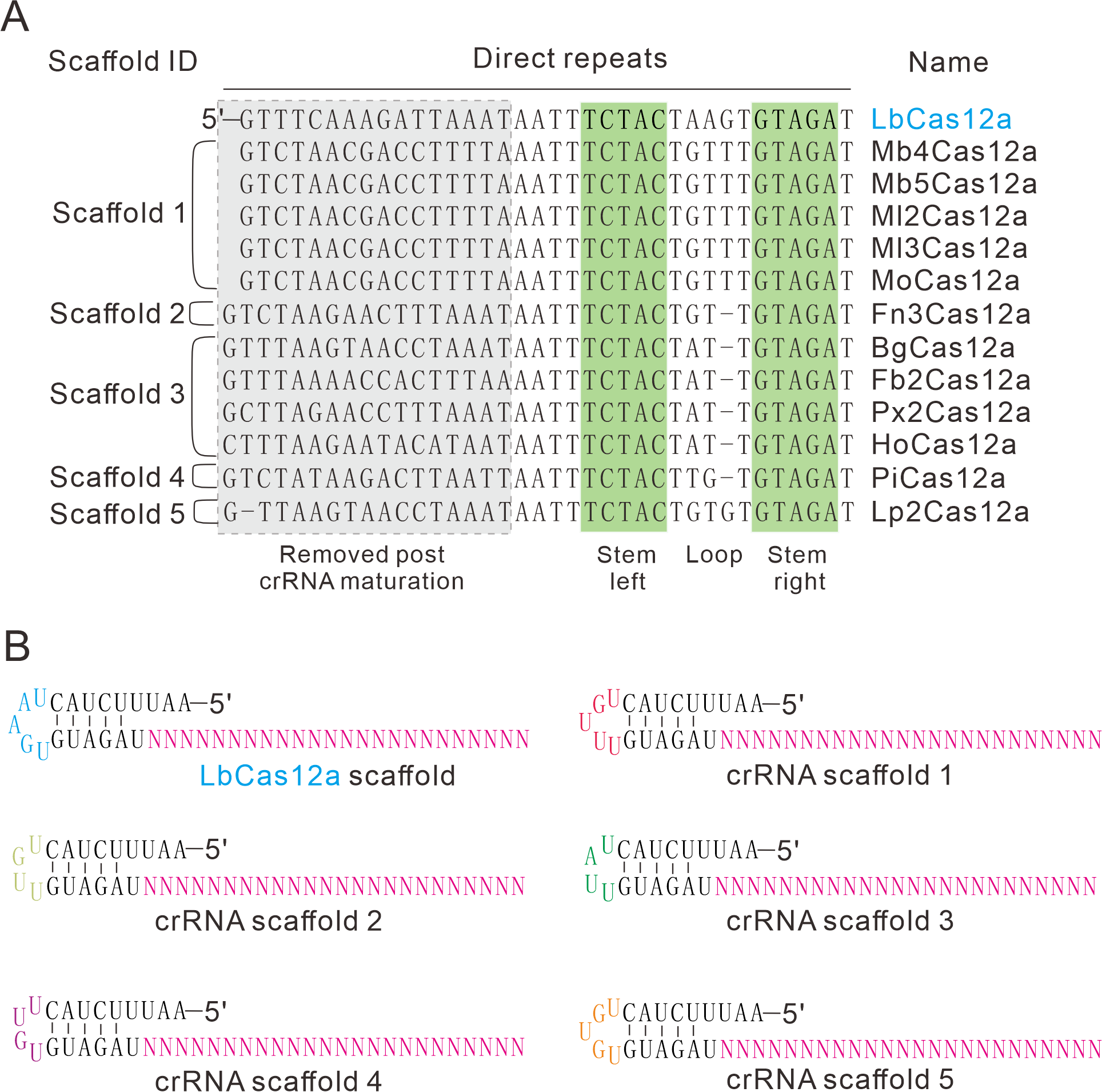

Supplement: S3 Fig — (A) Direct repeat sequences of 12 Cas12a orthologs. LbCas12a direct repeat sequences are used as a reference. Sequences removed post crRNA maturation are indicated by a grey background; Stem sequences are indicated by a green background. Mature crRNA sequences are divided into 5 groups based on loop sequences. (B) The second structure of mature crRNA scaffolds. (TIF) [file pbio.3002680.s003.tif]

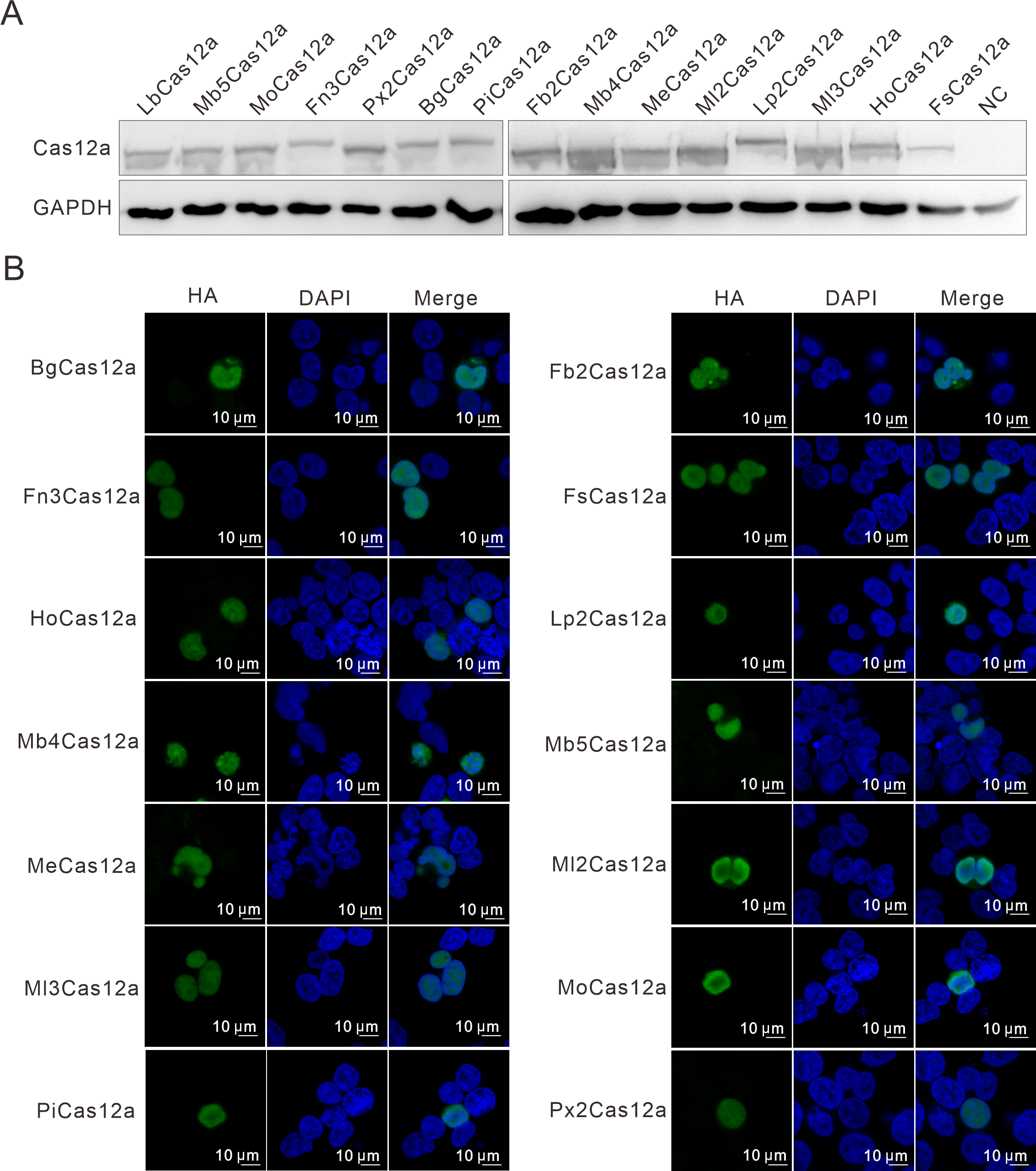

Supplement: S4 Fig — (A) Protein expression levels of each Cas12a ortholog are analyzed by western blot. HEK293T cells without Cas12a transfection are used as a negative control (NC). (B) The efficient nuclear localization of each Cas12a ortholog is confirmed by immunofluorescence staining. The cells are stained with anti-HA antibodies. Scale bar: 10 μm. (TIF) [file pbio.3002680.s004.tif]

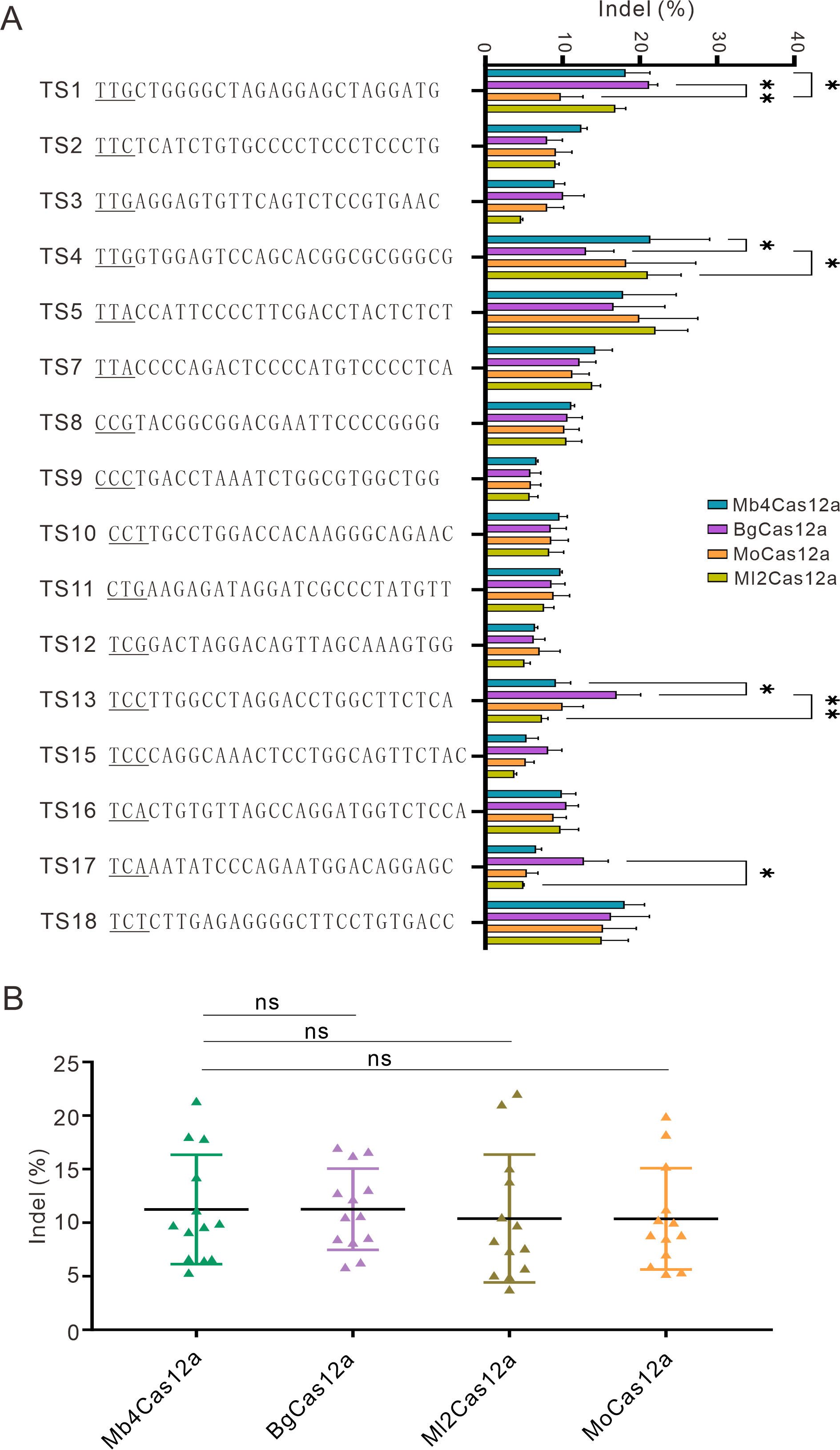

Supplement: S5 Fig — (A) Mb4Cas12a, BgCas12a, MoCas12a, and Ml2Cas12a enable genome editing with YYN PAMs in HEK293T cells. (B) Quantification of average editing efficiency for each Cas12a ortholog. ns stands for no significant. The numerical values underlying this figure can be found in S5 Table. The data represent the mean ± SD; n = 3. Two-way ANOVA, * p < 0.05. ** p < 0.01. ns means no significant. (TIF) [file pbio.3002680.s005.tif]

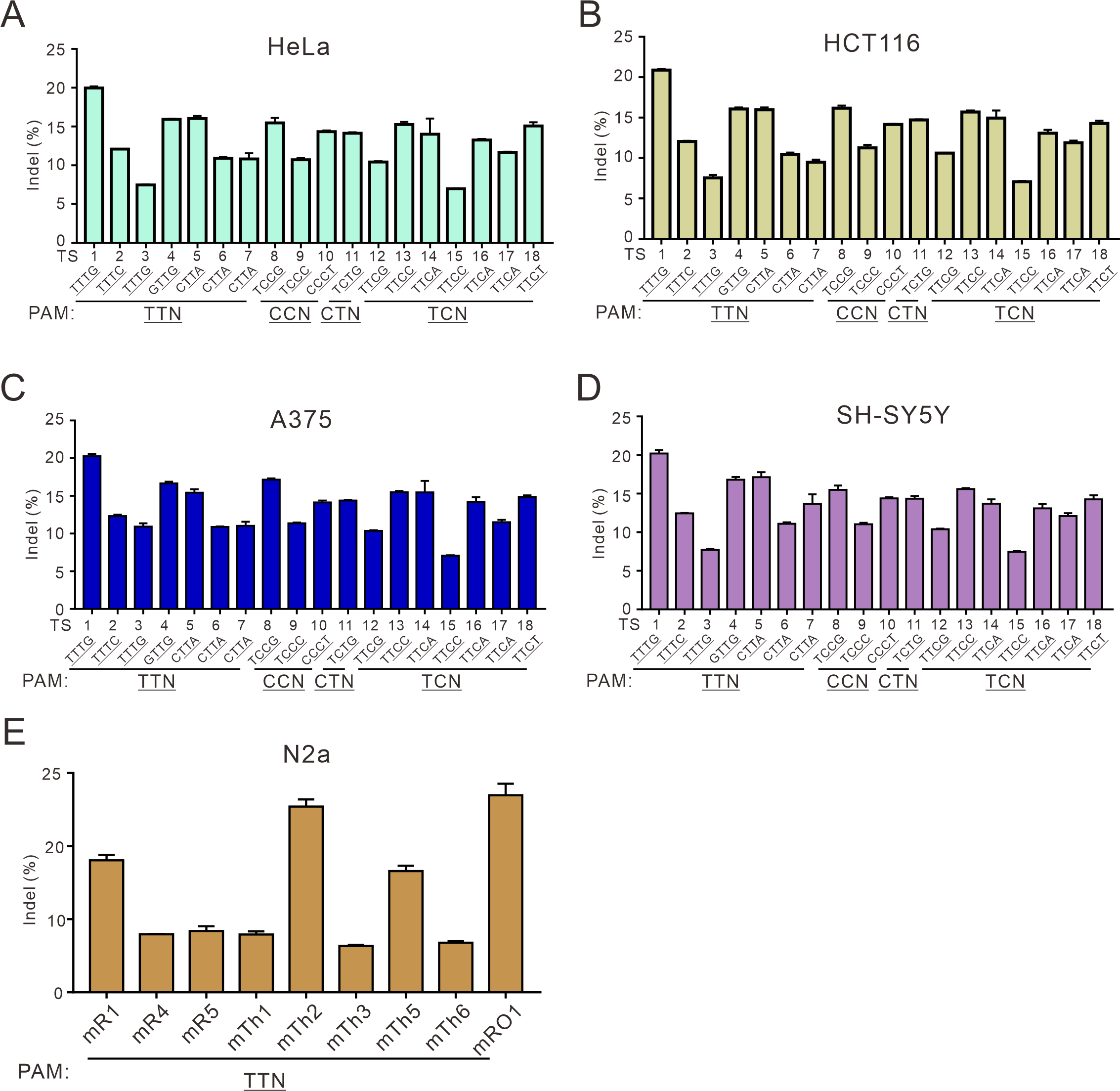

Supplement: S6 Fig — (A-E) Mb4Cas12a enables genome editing in HeLa (A), HCT116 (B), A375 (C), SH-SY5Y (D), and mouse N2a (E) cell lines. The numerical values underlying this figure can be found in S5 Table. (TIF) [file pbio.3002680.s006.tif]

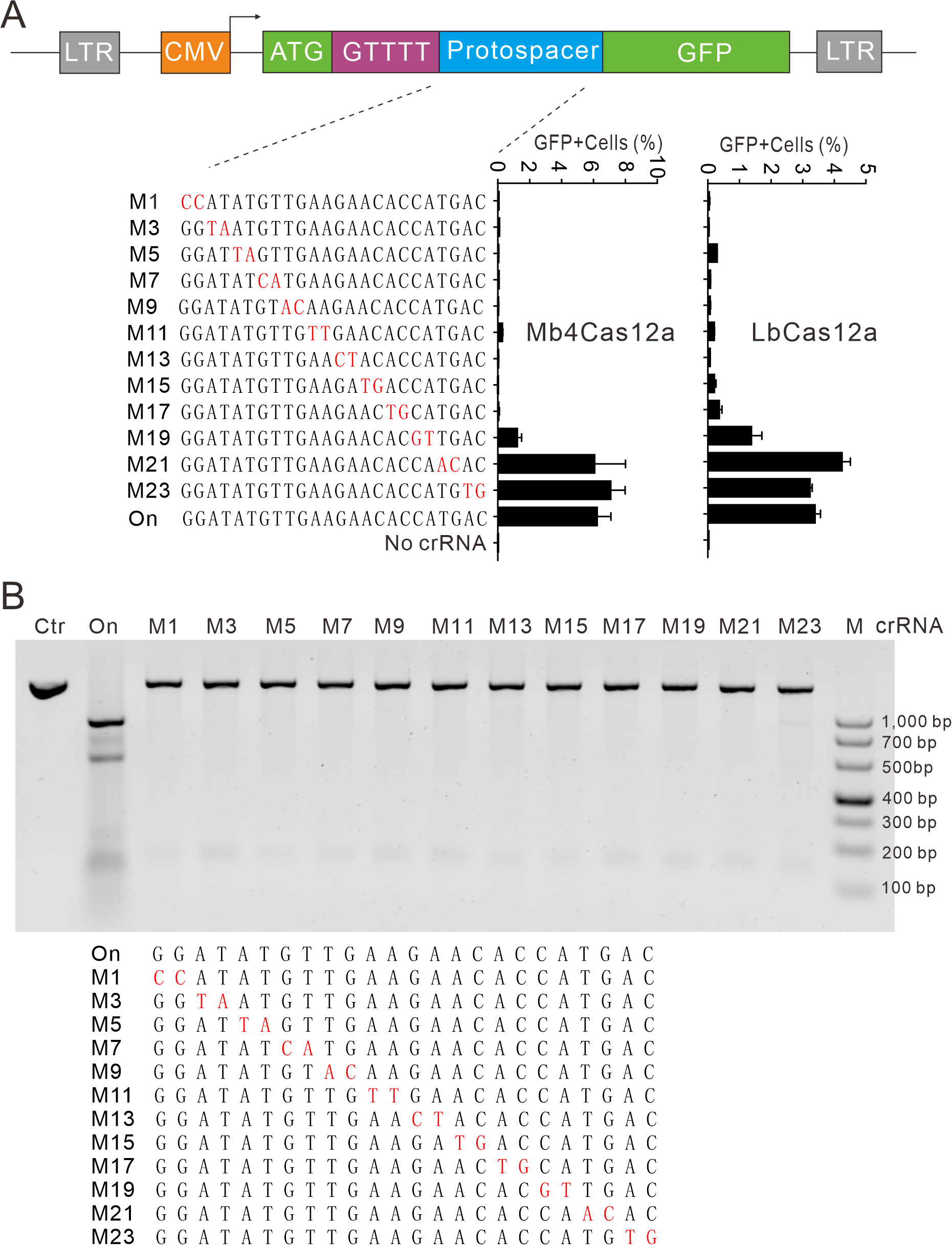

Supplement: S7 Fig — (A) The GFP-activation assay shows that Mb4Cas12a and LbCas12a are sensitive to the dinucleotide mismatches at crRNA positions 1–20. A schematic of the GFP-activation reporter is shown on the top; crRNAs are shown on the left; dinucleotide mismatches are indicated in red. The numerical values underlying this figure can be found in S5 Table. (B) Mb4Cas12a specificity is analyzed by the in vitro cleavage assay. Mb4Cas12a, crRNA, and DNA substrates are incubated at 37°C for 8 hours. crRNA sequences are shown below. Ctr, control, DNA substrates without digestion. On, on-target crRNA. (TIF) [file pbio.3002680.s007.tif]

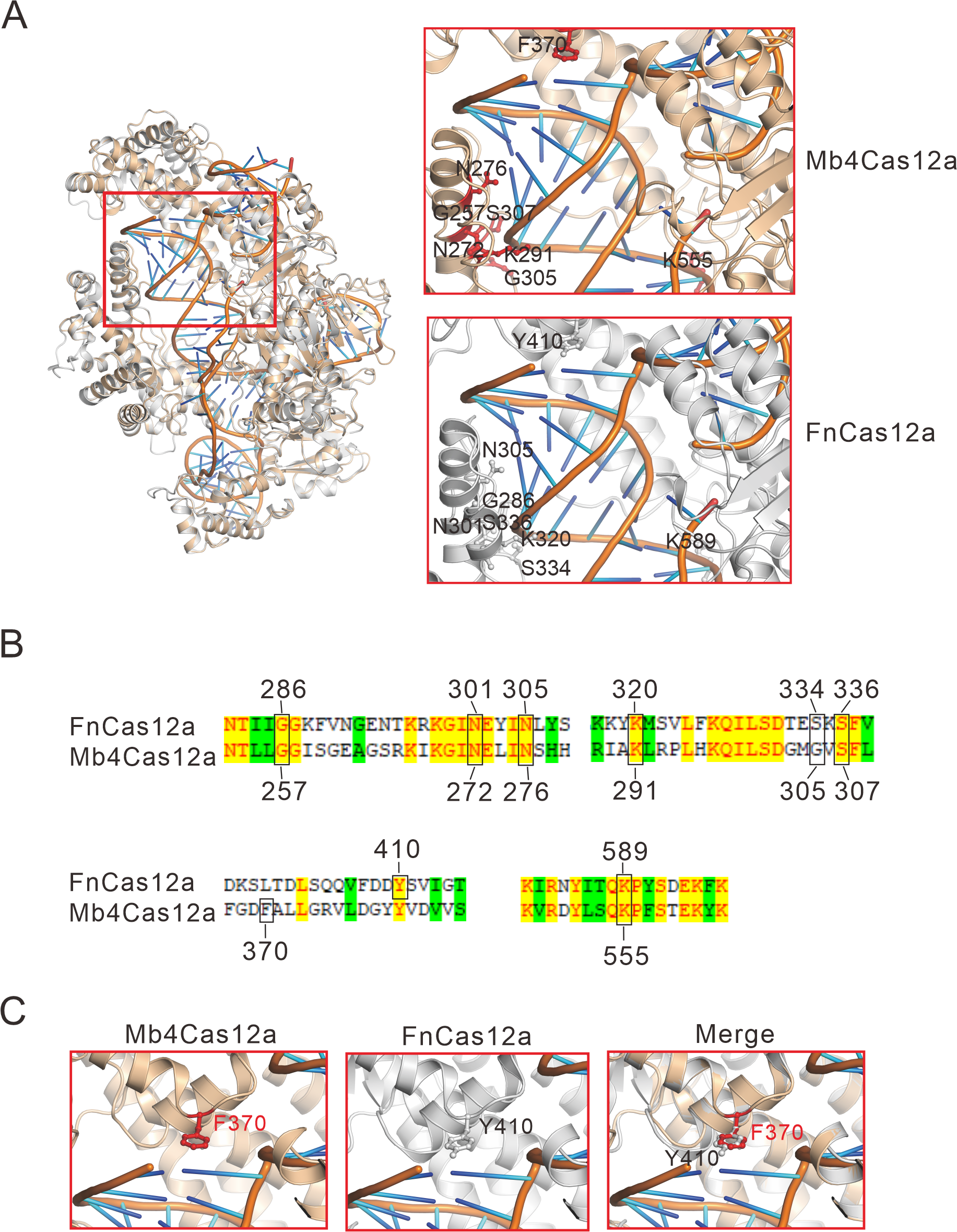

Supplement: S8 Fig — (A) Analysis of the FnCas12a crystal structure showed that 8 residues form hydrogen bonds at the target DNA-crRNA interface. (B) The corresponding residues of Mb4Cas12a are identified by protein sequence alignment to FnCas12a. (C) The predicted F370 of Mb4Cas12a is structurally consistent with Y410 of FnCas12a by SWISS-MODEL. (TIF) [file pbio.3002680.s008.tif]

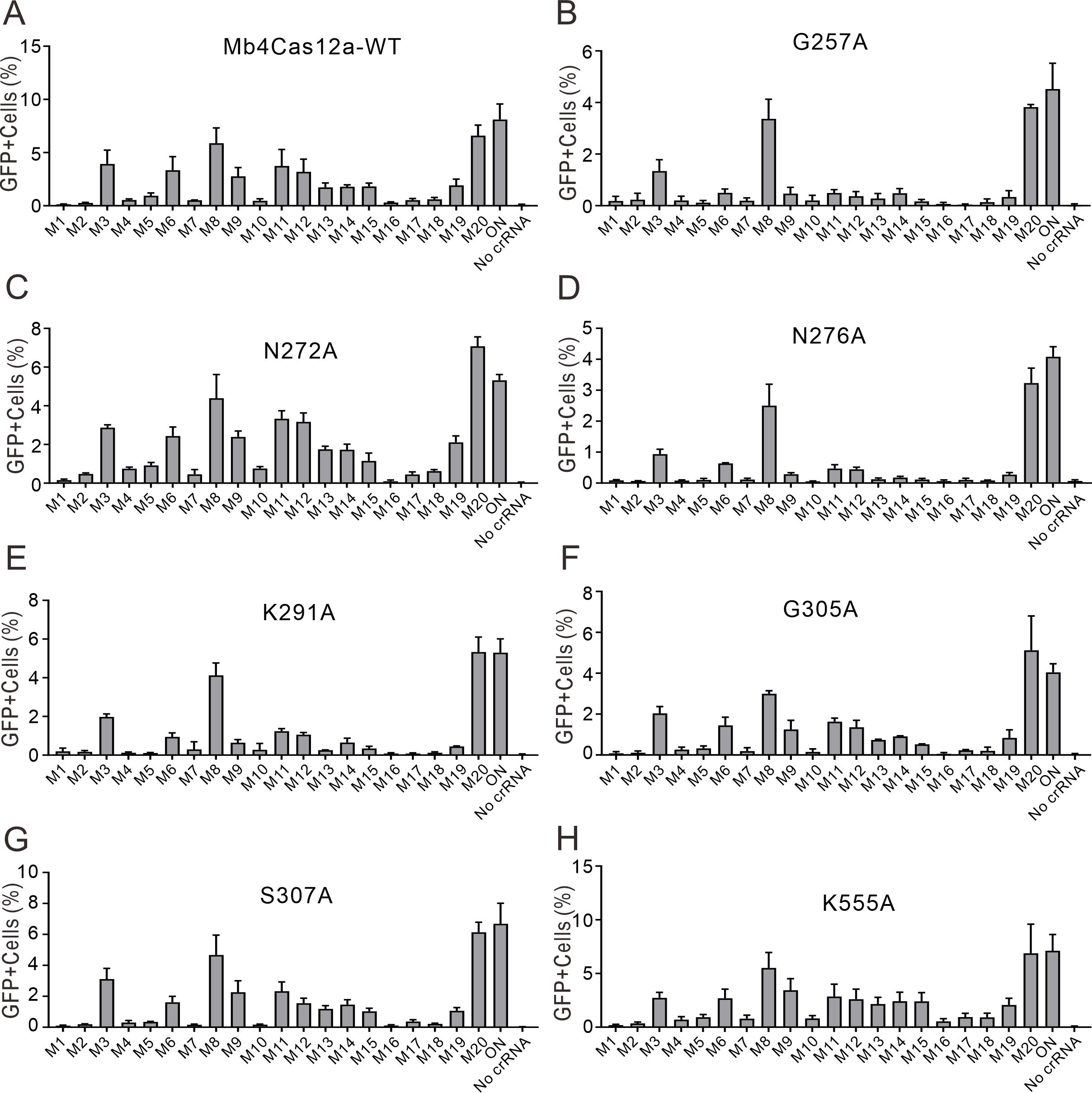

Supplement: S9 Fig — (A) The GFP-activation assay was used to evaluate the effects of Mb4Cas12a. (B-H) The GFP-activation assay was used to evaluate the effects of mutations G257A (B), N272A (C), N276A (D), K291A (E), G305A (F), S307A (G), and K555A (H) on Mb4Cas12a specificity. The numerical values underlying this figure can be found in S5 Table. (TIF) [file pbio.3002680.s009.tif]

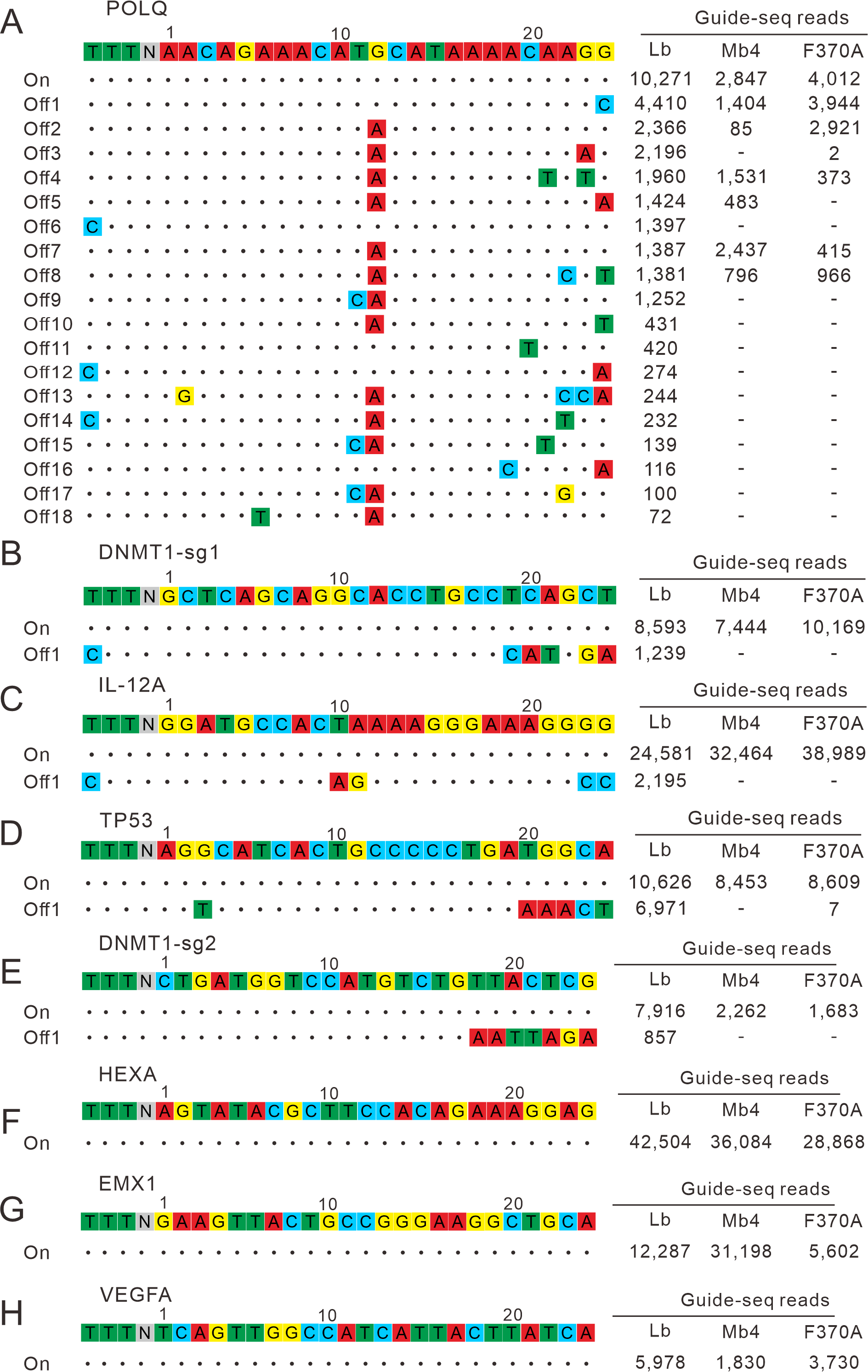

Supplement: S10 Fig — (A-H) The genome-wide off-target effects of LbCas12a, Mb4Cas12a, and F370A are analyzed by GUIDE-seq. On-target and off-target sequences are shown on the left. Read numbers for on-target and off-target sites are shown on the right. Mismatches compared to the on-target site are shown and highlighted in colour. (TIF) [file pbio.3002680.s010.tif]

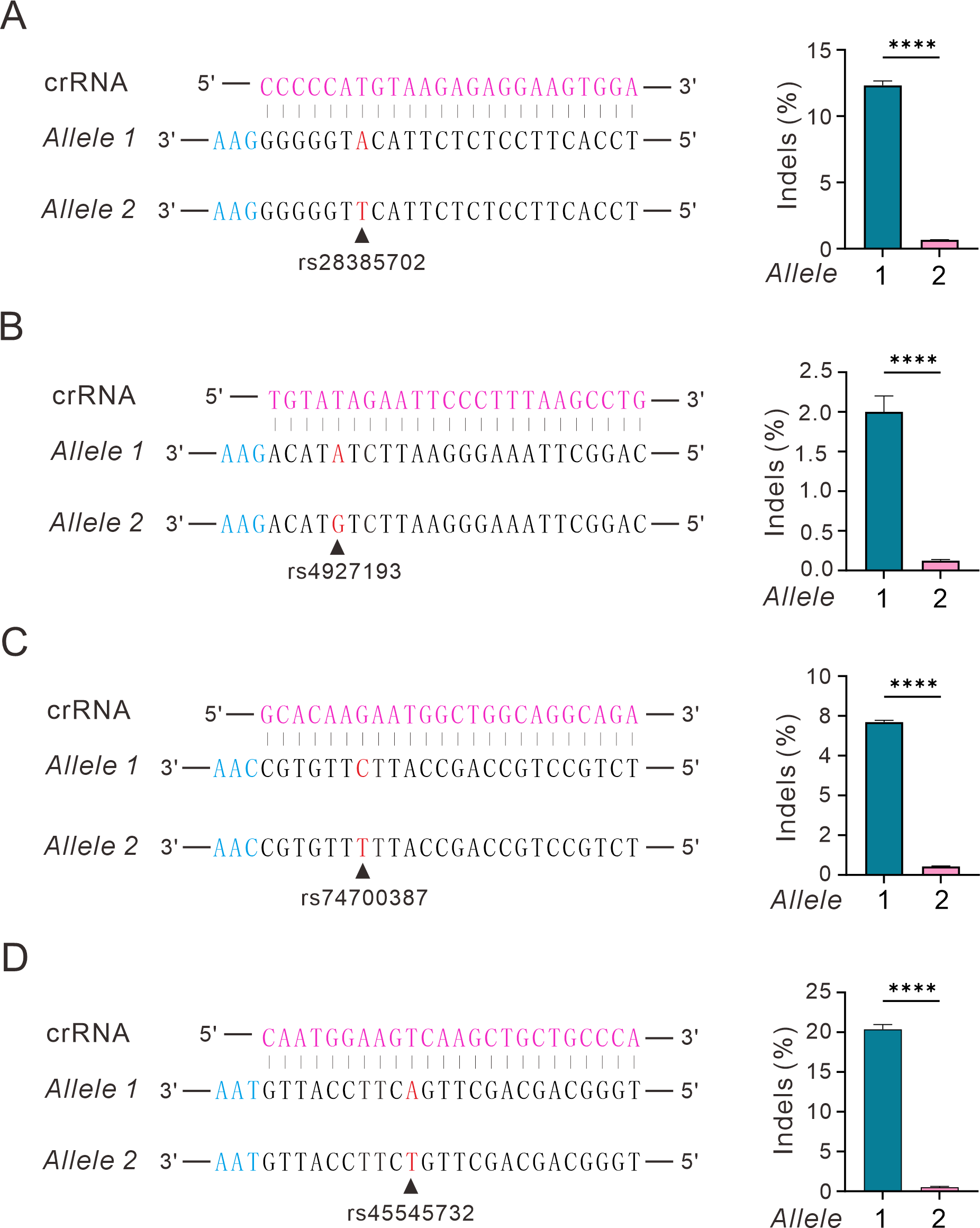

Supplement: S11 Fig — Mb4Cas12a-F370A allele specifically disrupts the single-nucleotide polymorphism (SNP) at locus rs28385702 (A), rs4927193 (B), rs747000387 (C), rs45545732 (D) in SH-SY5Y cells. PAMs are shown in light blue; target SNPs are shown in red; crRNAs are shown in purple; indel efficiencies are shown on the right. Data represent mean ± SD. n = 3. Student t test, **** p < 0.0001. The numerical values underlying this figure can be found in S5 Table. (TIF) [file pbio.3002680.s011.tif]

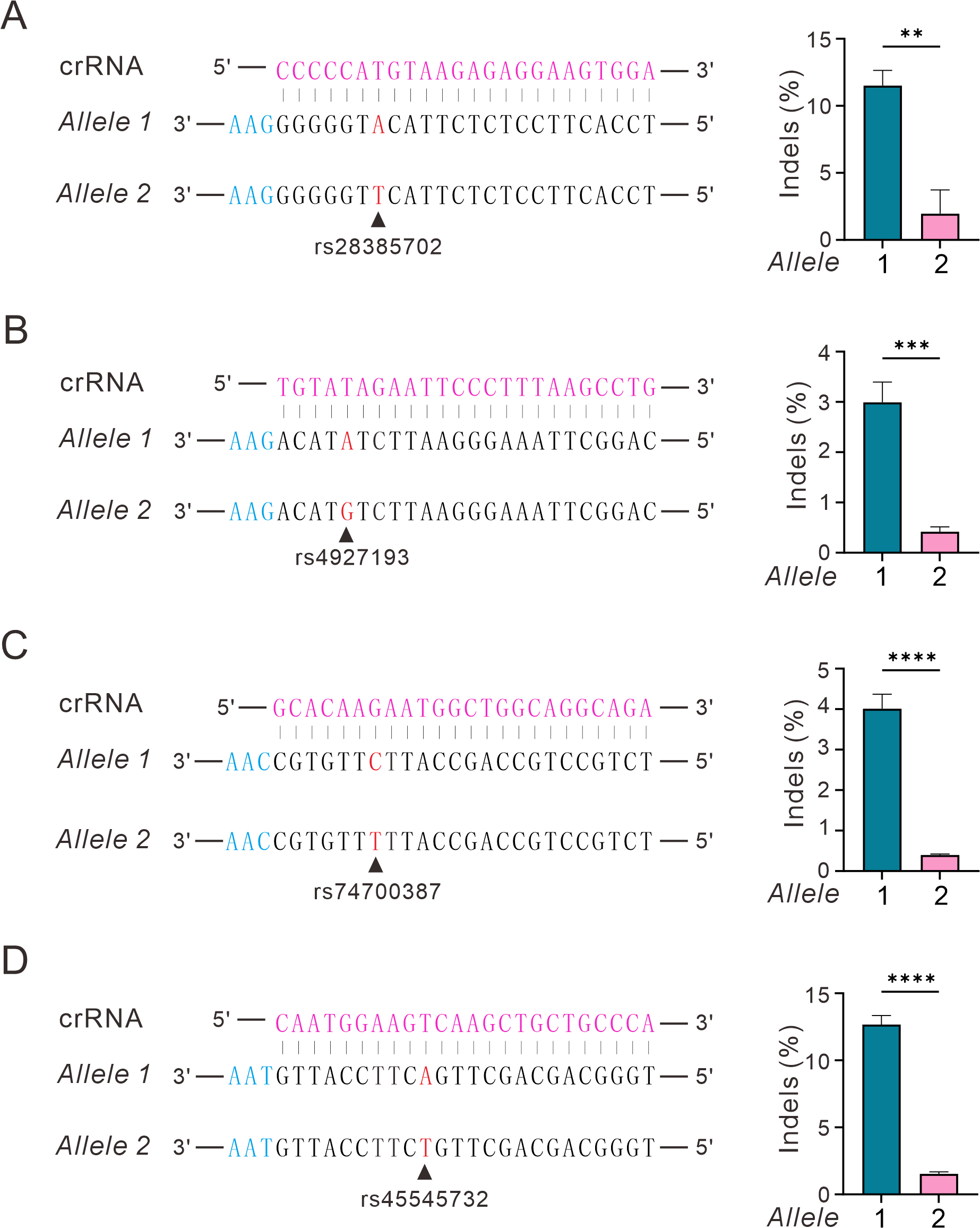

Supplement: S12 Fig — Mb4Cas12a-F370A allele specifically disrupts the single-nucleotide polymorphism (SNP) at locus rs28385702 (A), rs4927193 (B), rs747000387 (C), rs45545732 (D) in C33A cells. PAMs are shown in light blue; target SNPs are shown in red; crRNAs are shown in purple; indel efficiencies are shown on the right. Data represent mean ± SD. n = 3. Student t test, ** p < 0.01, *** p < 0.001, **** p <0.0001. The numerical values underlying this figure can be found in S5 Table. (TIF) [file pbio.3002680.s012.tif]
