# Supplementary material for: Engineering of a high-fidelity Cas12a nuclease variant capable of allele-specific editing
Source: PLoS Biol. 2024 Jun 12;22(6):e3002680. doi: 10.1371/journal.pbio.3002680 (PMC11168656; doi:10.1371/journal.pbio.3002680)

Corresponding to Figures 3A-B

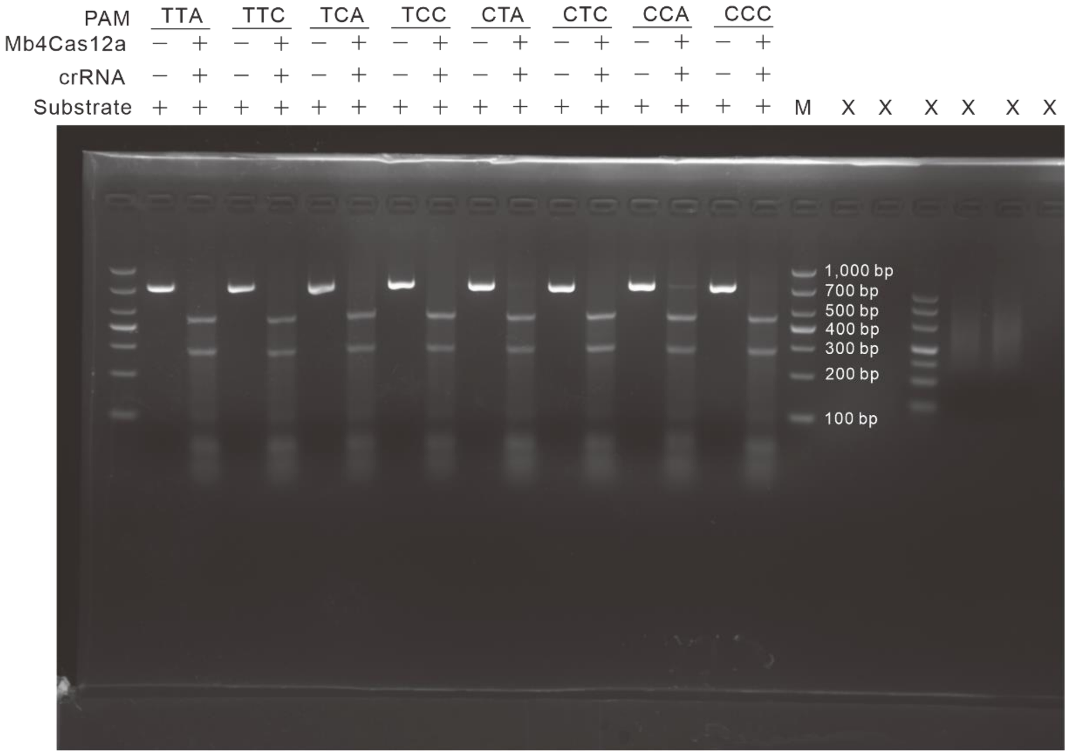

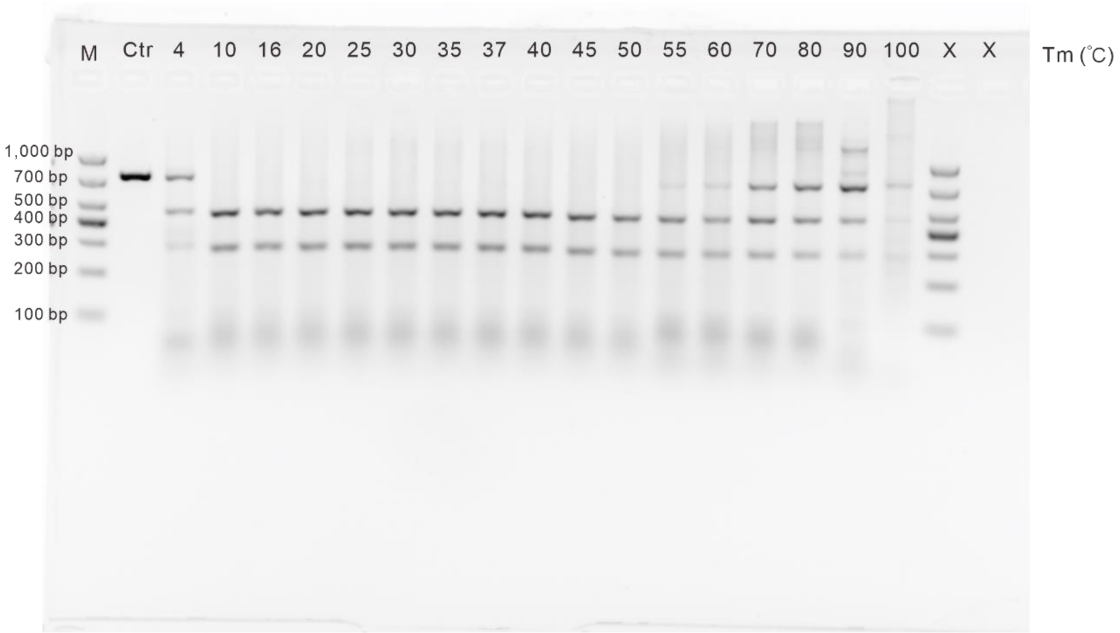

Corresponding to Figures S4A

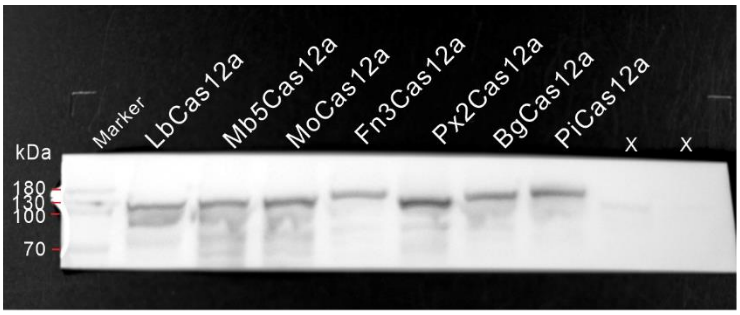

Cas12a-HA tag

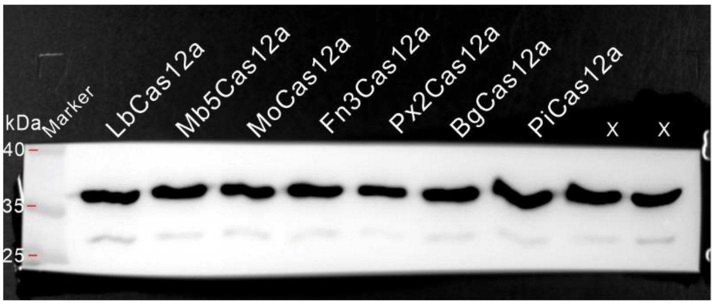

GAPDH

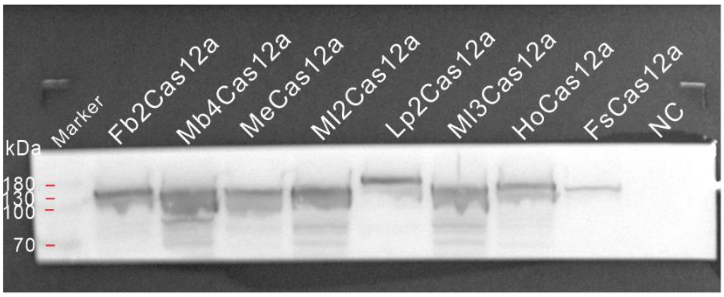

Cas12a-HA tag

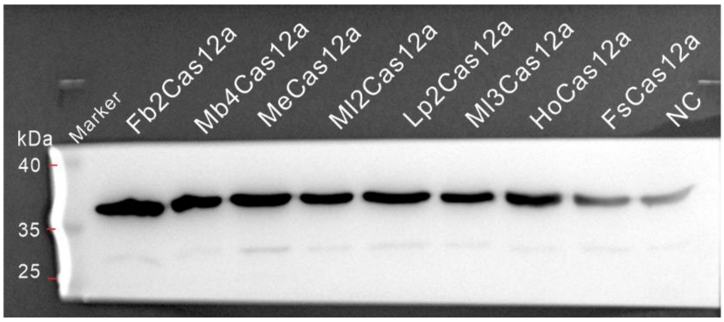

GAPDH

Corresponding to Figures S7B

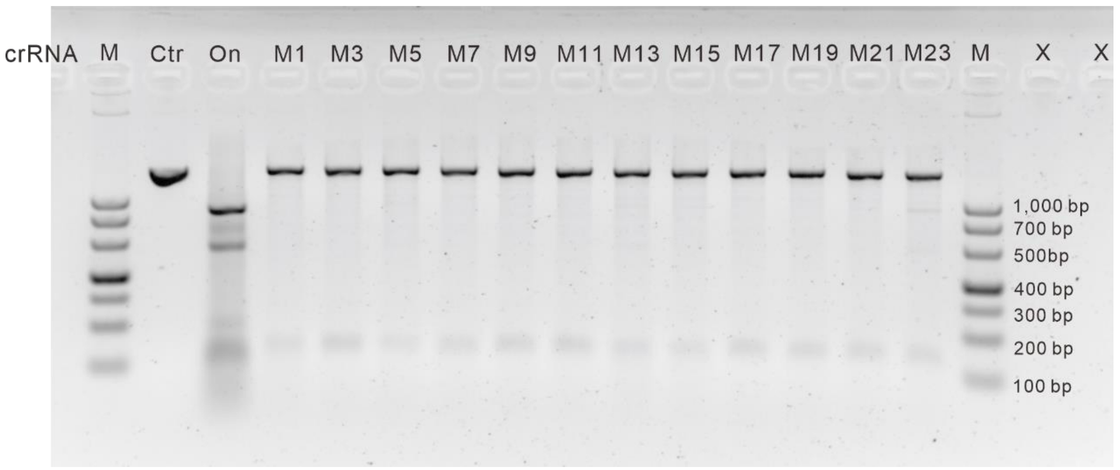

Corresponding to Figures 4B

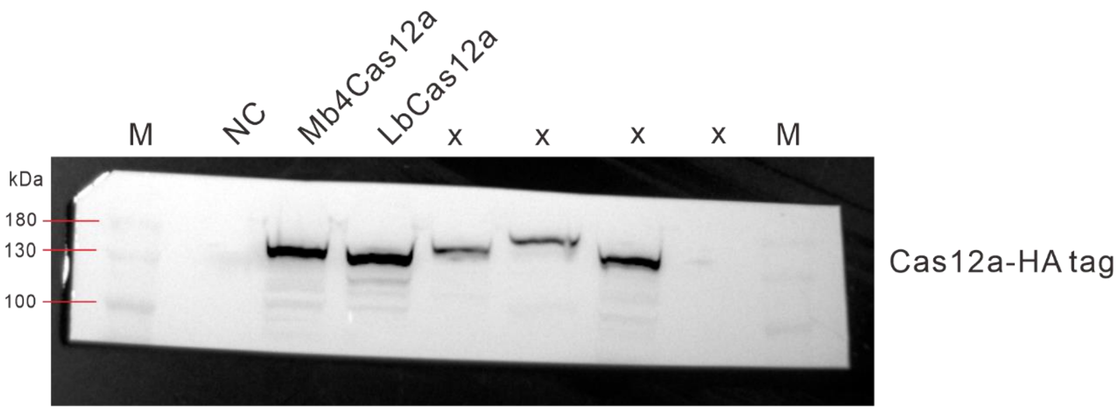

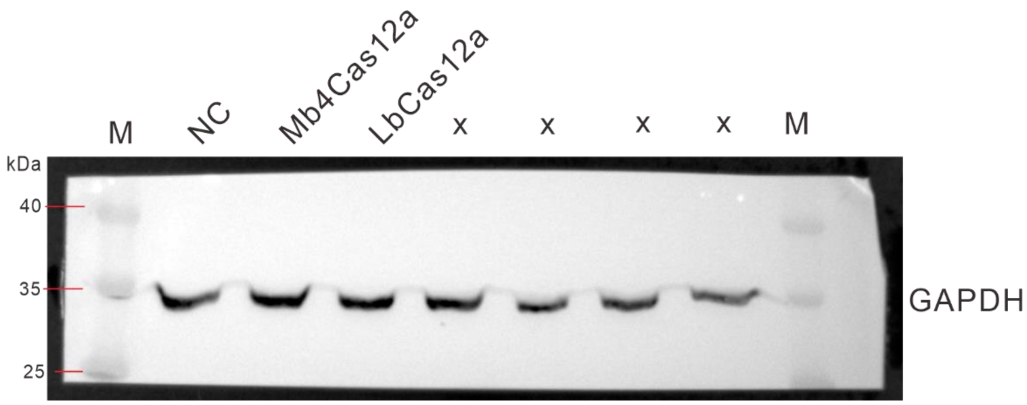

Supplement: S1 Raw Images — (PDF) [file pbio.3002680.s019.pdf]
