# Supplementary material for: Engineering of a high-fidelity Cas12a nuclease variant capable of allele-specific editing
Source: PLoS Biol. 2024 Jun 12;22(6):e3002680. doi: 10.1371/journal.pbio.3002680 (PMC11168656; doi:10.1371/journal.pbio.3002680)
Supplement: Attachment — Submitted filename: Response to Reviewers.docx [file pbio.3002680.s021.docx]

**Discovery and engineering of novel Cas12a nucleases for precision genome editing**

We express our sincere gratitude to the reviewers for their positive feedback and valuable suggestions to enhance our manuscript's quality. A copy of the comments (highlighted in grey) and a detailed point-by-point response can be found below. All changes and additions to the manuscript are marked for ease of review.

**REPLY TO EDITOR:**

1) As you will see, the reviewers think the study is well conducted but raise overlapping concerns with the strength of the biochemical characterization and overstatements in regards to SNP specificity. Specifically, Reviewer #2 notes that additional evidence for SNP specificity should be provided in at least one cell line of each lineage and that 4 independent loci are included in the analysis.

Reply: We sincerely thank the editor for the constructive comments on our manuscript. All three reviewers raised the concern about the specificity of single nucleotide polymorphisms (SNPs). While Reviewer #1 and Reviewer #3 suggested not emphasizing SNP specificity as the primary novelty, Reviewer #2 recommended either toning down the claim or presenting additional data related to cell lineage (ectoderm, mesoderm, and endoderm). In response to these valuable suggestions, we have adjusted the emphasis on SNP specificity in the revised manuscript. Moreover, to address the concern, we conducted additional experiments using four selected SNPs and tested Mb4Cas12a-F370A specificity in two cell lines representing ectodermal lineage (SH-SY5Y and C33A). The results, presented in Figure S12 and Figure S13, indicate that Mb4Cas12a-F370A can effectively discriminate these SNPs.

2) Reviewer #3 also notes that western blots should be provided for the orthologs tested in the GFP-activation assay to control for expression levels.

Reply: Thank you for your valuable suggestions. Following the reviewers' recommendations, we conducted Western blot analyses to verify the expression of each Cas12a protein (Fig. S5A).

**REPLY TO REVIEWER #1 (Public Review):**

The manuscript from W. J et al describes the discovery and engineering of novel Cas12a nucleases for precision genome editing. This work screened 14 Cas12a orthologs and characterized Mb4Cas12a in detail, and engineered Mb4Cas12a to improve specificity, enabling allele-specific editing of single-nucleotide polymorphisms (SNPs). The manuscript contains data on editing features in human cells and data on fidelity tests, as well as an incomplete biochemical characterization.

> Overall, the manuscript has both strengths and weaknesses. Strengths include that new Cas12a systems were characterized, broadening our understanding of active Cas12a nucleases. Weakness is that the quality and reliability of the data need to be improved, and the manuscript has some flaws in writing.

> Weaknesses:

> 1.	In Fig 3A, the cleavage substrates of various PAMs belong to different target sites. It seems not convincing to compare PAM preference at different sites in vitro, due to the Cas nuclease may have site specificity, and the conclusions drawn from the cleavage assay are questionable.

Reply: Thank you for your comments. Following the reviewer's suggestion, we designed a target with different PAMs. The in vitro cleavage assay shows that Mb4Cas9 can efficiently cleave the target with TTA, TTC, TCA, TCC, CTC, and CCC PAMs while less efficiently cleave the target with CTA and CCA PAMs (Figure 3A).

Figure 3A. DNA cleavage of Mb4Cas12a at 37 °C for eight hours.

> 2.	Fig 6 shows the data of GUIDE-seq. the reads number of the Mb4Cas12a and mutant F370A is much less than that of LbCas12, and the difference in 4 of 6 sites (Fig 6A-C, 6F) even reach to 10-fold. Whether this obvious difference results from the editing activity at these target sites or from experimental manipulation needs plausible explanation.

Reply: Thank you for your suggestions. Following the reviewer's suggestions, we tested the editing activity of these target sites. The results showed that the efficiency difference between these Cas12a orthologs was less than 1.8 times at these sites (Fig. S10). Notably, the on-target reads number of LbCas12a, Mb4Cas12a, and F370A varied dramatically, probably due to experimental manipulation during deep sequencing. To fairly compare their off-target effects, we took a comparable number of reads to analyze the off-target effects. The results showed that LbCas12a induced 27 off-targets across six tested loci. In contrast, Mb4Cas12a, induced six off-targets across six tested loci, while F370A generated seven off-targets across six tested loci (Fig. S11). These data indicated that Mb4Cas12a and F370A were more specific than LbCas12a.

Figure S10. The indel efficiency of LbCas12a, Mb4Cas12a, and F370A.

Figure S11. Test the Mb4Cas12a specificity with GUIDE-seq with an equal number of reads.

> 3.	The engineered Mb4Cas12a mutant F370 does not correspond to the sequence alignment in the SI figure. Fig S6B shows the 370 AA of Mb4Cas12a is Y.

Reply: We are sorry for the incorrect annotation. In the revised manuscript, we corrected the incorrect annotations in Fig S8. SWISS-MODEL of Mb4Cas12a showed that F370 instead of Y381 corresponded to FnCas12a Y410 (Fig S8C).

Figure S8. Identification of residues that potentially form hydrogen bonds.

> 4.	The paper does not explain why alanine mutants of the amino acid which interact with DNA-crRNA duplex could improve Mb4Cas12a specificity.

Reply: Thank you for your suggestions. In the revised manuscript, we explained why alanine mutants of the amino acid could improve Mb4Cas12a specificity as follows:

A previous study has hypothesized that the SpCas9-sgRNA complex may possess more energy than is required for optimal recognition of its intended target DNA site, potentially facilitating the cleavage of mismatched off-target sites. This phenomenon is partially attributed to certain Cas9 amino acid residues forming hydrogen bonds with the target DNA. Disrupting these hydrogen bonds has been shown to increase the specificity of Cas9.

> 5.	The mutant of Mb4Cas12a had the ability to identify SNP sites and make selective editing, and the authors had a similar presentation: A highly specific CRISPR-Cas12j nuclease enables allele-specific genome editing (Science Advances). What would be the advantage of Cas12a reported in this manuscript over the previously reported Cas12j system?

Reply: Thank you for your comments. In comparison to Cas12j, which recognizes a TTN PAM, Mb4Cas12a identifies a YYN PAM, thus broadening the targeting scope. In addition, each Cas nuclease exhibits a distinct target sequence preference, offering an opportunity to edit more genomic sites.

> 6.	The notes in Fig1C and Fig 4C mentioned Cas9, which seems a mistake in writing.

Reply: We apologize for the error in our writing. In the revised manuscript, we corrected "Cas9" to "Cas12a".

**REPLY TO REVIEWER #2 (Public Review):**

> Reviewer #2: The manuscript submitted by Wei et al. titled "Discovery and engineering of novel Cas12a nucleases for precision genome editing" describes the characterization and functional analysis of several programmable endonucleases which are part of the Cas12a family. In this study, the authors focus on fourteen Cas12a orthologs and test their activity in vitro and in vivo using a variety of human cell lines. Wei and colleagues find that one of the orthologs, Mb4Cas12a, outperforms the other Cas12a variant tested with respect to on-target DNA cleaving activity and specificity (i.e. less off-target activity, more sensitive to mismatches). The latter motivates the authors to investigate whether the specificity and activity of Mb4Cas12a can be further improved by mutating eight highly conserved amino acid residues that potentially form hydrogen bonds at the target DNA-crRNA interface (based on previously published crystal structure data of FnCas12a). The authors show that the engineered mutant Mb4Cas12a-F370A outperforms both Mb4Cas12a and the reference LbCas12a in terms of specificity. In addition, the authors show to some extent that Mb4Cas12a-F370A is able to discriminate between alleles harbouring single nucleotide polymorphisms ("mismatches") and could potentially be further developed to treat autosomal dominant diseases through allele-specific disruption of mutant alleles.

> Although the data presented in the manuscript is generally worked out well and with attention to detail, some figures require additional data to justify the scientific claims made by the authors.

> These specific issues are described in more detail below.

> Major issues:

> - The authors claim that allele-specific editing is possible using an engineered version of Mb4Cas12a. However, allele-specific editing of the rs6684195 locus resulted in an indel rate of 28.3% at the target allele and 2.0% at the non-target allele. Although this is an almost 15-fold difference, one cannot claim that this method is specific. This difference is even smaller for the locus rs28385702 (indel rate: 5% at the target allele and 0.8% at the non-target allele). In addition, the authors provide data for only two genomic targets analyzed for allele-specific editing in a single cell line (HEK293T). However, it remains unclear whether allele-specific editing is supported in other cell types as well. Together, this is insufficient evidence to claim that the new Cas12a variants enable allele-specific editing in human cell lines. Therefore, the authors need to either adjust their claim about Mb4Cas12a-mediated allele-specific editing or provide more supporting evidence showing at least similar fold differences in at least one cell line of each lineage (ectoderm, mesoderm, endoderm).

Reply: Thank you for your valuable comments. All three reviewers raised concerns about the allele-specific editing. Reviewer #3 suggested not emphasizing allele-specific editing as the primary novelty. In response to these valuable suggestions, we have adjusted the emphasis on SNP specificity in the revised manuscript. Moreover, to address the concern, we conducted additional experiments using four selected SNPs and tested Mb4Cas12a-F370A specificity in two cell lines representing ectodermal lineage (SH-SY5Y and C33A). The results, presented in Figure S12 and Figure S13, indicate that Mb4Cas12a-F370A can effectively discriminate these SNPs.

Figure S12. Mb4Cas12a-F370A allows allele-specific genome editing in SH-SY5Y cells.

Figure S13. Mb4Cas12a-F370A allows allele-specific genome editing in C33A cells.

In addition, the authors need to show that allele-specific editing is possible in at least four independent loci (ideally, randomly chosen from the 3084 clinically relevant variants listed in Table_S4). This is in line with previous work by Wang et al. - DOI: 10.1126/sci adv abo6405 (2023) wherein allele-specific genome editing by Cas12j is described.

Reply: Thank you for your valuable suggestion. We acknowledge the importance of editing clinically relevant variants, but unfortunately, we currently lack cell lines containing these mutations. The creation of such cell lines would require more time than our revision period allows. Therefore, we have opted to select additional SNPs for editing in the revised manuscript (Figure S12-S13).

> Minor issues:

> - Sanger sequencing and NGS data for any of the experiments described in the manuscript are not included and therefore need to be provided to be able to verify the results.

Reply: We thank the reviewer for the comments. Following the reviewer's suggestions, the information on raw sequencing data is listed in Table_S6. The raw sequencing data have been submitted to the NCBI Sequence Read Archive (SRA PRJNA: 1055423 (https://dataview.ncbi.nlm.nih.gov/object/PRJNA1055423?reviewer=76v3nvvrmefta14undti82dun2).

> - The methods section provides very little information for several key experiments or is missing altogether. For example, it is unclear how phylogenetic analysis was performed for Figure S1.

Reply: Thank you for your suggestions. In the methods section, we provided information for phylogenetic analysis as follows:

Phylogenetic analysis was conducted using the vector NTI software, employing its default parameters. The Clustal W algorithm served as the alignment method. In essence, an initial assessment of crude similarity among all sequence pairs, termed "Parities alignment," is conducted. These similarity scores are subsequently employed in generating a "guide tree" or dendrogram. This dendrogram dictates the order in which sequences are aligned during the final multiple-alignment stage. Following the calculation of the dendrogram, sequences are aligned progressively, starting with smaller groups and expanding until the entirety of sequences is encompassed in the conclusive alignment. This method ensures a systematic and comprehensive approach to multiple sequence alignment.

In addition, we also provided information for the construction of the GFP activation system, PAM sequence analysis, and immunofluorescence staining.

Also, it is unclear how the GUIDE-Seq methodology was performed and the data analyzed.

Reply: Thank you for your suggestions. We added details in the revised manuscript as follows:

"500 ng of genomic DNA from each sample were randomly fragmented by NEBNext Ultra II FS DNA Module (NEB). A cleanup with 35 μL of AMPure XP beads (1X ratio) is performed according to manufacturer protocol, and eluted in 12 μL of ddH2O. 10 μL end-repaired DNA was incubated with 10 μL NEBNext Adapter (E7335L), and 5 μL Quick T4 DNA Ligase in 2x Quick Ligation Reaction Buffer at 20 °C for 15 min. The sequence of NEBNext Adapter is listed as follow: 5´-/5Phos/GAT CGG AAG AGC ACA CGT CTG AAC TCC AGT CdUA CAC TCT TTC CCT ACA CGA CGC TCT TCC GAT C-s-T-3´. Then 3 µl of USER® Enzyme was added to the ligation mixture at 20 °C for 15 min. Two rounds of nested anchored PCR, with primers complementary to the oligo tag, were used for target enrichment. Amplicons were purified by AMPure XP beads and sequenced on an Illumina sequencer. Restriction fragment-length polymorphism (RFLP) assays were used to assess oligo tag integration rates. The primer sequences for Guide-seq are listed in Table_S2".

Finally, it is unclear how the GFP reporter cell line used to assess PAM specificity was generated and characterized. Please provide details.

Reply: Thank you for your suggestions. The details were added in the revised manuscript as follows.

**Construction of GFP activation system for PAM detection**

The reporter plasmid was constructed as previously described with minimal modifications [1]. Briefly, The DNA oligonucleotide (NNNNNGGATATGTTGAAGAACACCATGAC) with 20-nt flanking homologous sequences (AAGCCTTGTTTGCCACCATG / GTGAGCAAGGGCGAGGAGCT) for Gibson Assembly, were synthesized by GENEWIZ (Suzhou, China). Subsequently, full-length oligonucleotides were PCR-amplified utilizing Q5 High-Fidelity 2X Master Mix (NEB), followed by size selection on a 3% agarose gel EX (Life Technologies, Qiagen) and purification using the MinElute Gel Extraction Kit (Qiagen). The PCR products were then cloned into a lentiviral vector through Gibson Assembly (NEB) and further purified with Agencourt AMPure XP SPRI beads (Beckman Coulter). Electroporation of the Gibson Assembly products into MegaX DH10BTM T1R ElectrocompTM Cells (Invitrogen) was carried out using a GenePulser (BioRad). Subsequently, the bacteria were introduced into recovery media and allowed to grow at 32°C with agitation at 250 rpm overnight. Plasmid DNA was then purified from the bacteria using the Endotoxin-Free Plasmid Maxiprep kit (Qiagen).

**PAM sequence analysis**

The reporter plasmids were packaged into lentivirus and transfected into HEK293T cells as previously described [1]. Five days after transfection of Cas12a (10 μg) and gRNA-expressing plasmids (5 μg) into cells, genomic DNA was isolated for targeted deep sequencing of the random region. For PAM analysis, the 20-nt oligonucleotide flanking the target sequence was used to fix the target sequence. The sequences TTGTTTGCCACCATG and GGA were employed to fix the 5-nt random sequence. Target sequences with in-frame mutations were utilized for PAM analysis. The 5-nt random sequence was extracted and visualized using WebLogo3 [2] and a PAM wheel chart [3] to illustrate PAMs.

> - Figure 1 and 4 read "The cells without transfection of Cas9 were used as a negative control", but I assume the authors meant Cas12a.

Reply: We are sorry for the mistake in writing. In the revised manuscript, we changed it to Cas12a.

> - For Figures S7 and S8, please include data from WT Mb4Cas12a as a comparison. NB: I would suggest the authors combine these two figures into one.

Reply: Thank you for your suggestions. In the revised manuscript, we added the data from WT Mb4Cas12a and combined Figures S7 and S8 into Figure S9.

Figure S9. Effects of seven single mutations on Mb4Cas12a specificity.

> - The data in Table S4 is shifted from row 441 onwards. Please modify.

Reply: Thank you very much for your suggestions. In the revised manuscript, we modified the shift in Table S4.

> - The names of the tables listed on the last page of the supplementals do not correspond with the files; Table_S3 is missing from the list and Table_S5 does not seem to exist (nor is it mentioned in the main manuscript).

Reply: Thank you very much for your comments. In the revised manuscript, we corrected the layout of the tables.

**REPLY TO REVIEWER #3 (Public Review)**:

> Reviewer #3: The authors of this manuscript describe the characterization and application of new Cas12a nucleases. Using sequence comparisons, the authors start by identifying several new Cas12a proteins originating from various bacteria species. Using a GFP activation assay, the authors characterize the PAM sequence preference of all the tested variants. They then pursue characterization of both the efficiency and fidelity of the most promising variant: Mb4Cas12a (originating from Moraxella bovis). The authors subsequently generated mutated versions of Mb4Cas12a in order to improve its fidelity and validated that the mutations contribute towards this improvement.

>

> Continuous characterization of Cas enzyme variants is crucial for the improvement of CRISPR/Cas based technologies. The various advancements that scientists are working on include increased efficiency, specificity, and flexibility (in the context of PAM preferences). The manuscript submitted by Wei et al. aligns with this overarching goal, as they explore new Cas12a variants, and therefore, it is timely, important, and has the potential for some impact. However, the novelty that the authors emphasize in this work about the SNP specificity of their new Cas12a variant is overstated as other Cas12a variants have been shown in literature to be highly specific and capable of discriminating SNPs. Aside from that, we believe the work presented in this manuscript is valuable to the scientific community and should eventually be published. However, we cannot recommend publication of the manuscript as it is currently constructed. Below we provide commentary, questions, and suggested experiments for the authors. These are intended to strengthen the authors' conclusions and focus the text to highlight novel and critical information. Finally, we note that it would be very helpful to include line numbers in any future iterations of this manuscript, as referring to specific text in their absence is tedious for reviewers.

>

> Major Points

>

> 1) Introduction: The authors mention: "A handful of Cas12a nucleases have been developed for genome editing, but none of them can discriminate single-nucleotide polymorphism (SNP)" as well as "Although Cas12a displays high specificity, they tolerate single or double mismatches". This claim seems overstated - for example, AsCas12a Ultra (PMID 34162850) shows excellent avoidance of off-target cutting (we recognize that this is not exactly the same as a demonstrated ability to distinguish SNPs). Additionally, there are numerous examples of Cas12a losing nearly all activity on the basis of a single point mutant. As we noted above, we don't think SNP detection is an important deliverable of this manuscript, so we highly encourage the authors not to overplay the novelty of their claims in this area.

Reply: We sincerely appreciate your valuable suggestions. We did not emphasize SNP detection as the primary novelty in the revised manuscript.

> 2) Results - Screen of active Cas12a orthologs by the GFP-activation assay: It appears that the sequence identity percentages between the various Cas12a orthologs and LbCas12a in Table 1 are relatively low. A sequence comparison of the functional domains could be more informative.

Reply: Thank you for your suggestions. Following the reviewer's suggestion, we compared each functional domain of new orthologs to LbCas12a. Further analysis of each function domain showed that the WED domain (playing a key role in crRNA recognition and processing) and the RuvC endonuclease domain had a higher identity to LbCas12a (Fig. S1A-B).

Figure S1. The comparison of functional domains.

> 3) Results - Screen of active Cas12a orthologs by the GFP-activation assay: After transfection of the various Cas12a variants in the cells, the authors measure GFP expression as a readout for editing efficiency (Fig1C). To rule out that the difference in performance is not due to differences in levels of expression the authors should perform western blots for all the orthologs tested. The authors should also address the efficient nuclear localization of these Cas12a variants by staining or by performing nuclei specific western blot.

Reply: We appreciate the valuable feedback from the reviewer, prompting us to perform Western blot analyses to assess the expression levels of each Cas12a protein. Despite utilizing the same construct backbone for their expression, these Cas12a orthologs exhibited varying protein expression levels (Fig. S4A). Immunofluorescence staining confirmed the nuclear localization of each Cas12a ortholog (Fig. S4B). Notably, FsCas12a exhibited the lowest protein expression, approximately 70% of LbCas12a expression, while Fx2Cas12a displayed the highest expression, reaching about twice that of LbCas12a. However, neither FsCas12a nor Fx2Cas12a demonstrated any activity in human cells. The remaining Cas12a orthologs exhibited minor expression variations, which may contribute, in part, to the observed variations in activity.

Figure S4. The verification of Cas12a orthologs expression level.

> 4) Results - Analysis of Mb4Cas12a specificity: To increase Mb4Cas12a specificity, the authors identify critical amino acid residues within the protein to mutate. They rely on the crystal structure of FnCas12a and compare its sequence to that of Mb4Cas12a to generate residue mutations. So far LbCas12a was the reference in this manuscript, can the authors explain why they chose FnCas12a here? How do these mutations compare to the extensive mutagenesis performed in FnCas12a by Liu et al., 2021?

Reply: We thank the referee to point out this issue. To generate a proper structural model for identifying critical amino acid residues involved in dsDNA binding, we need an existing Cas12-crRNA-dsDNA ternary structure as a template for Mb4Cas12a. However, currently, there is no experimental structure of the LbCas12a-sgRNA-dsDNA complex in the database. The existing LbCas12a-related structures are only with crRNA but lack the dsDNA target (and resolutions of these resolutions are quite low). For the Cas12-crRNA complex, the loading of the dsDNA substrate usually causes a large conformational change in the protein. Therefore, though ideally, LbCas12a should be used as a structural reference for Mb4Cas12a for consistency, we were unable to find a reliable LbCas12a-crRNA-dsDNA ternary complex for this purpose. We then chose an experimental FnCas12a-crRNA-dsDNA ternary complex with a decent resolution of 2.65 Å, which showed the highest Swiss-Model GMQE (Global Model Quality Estimate) value among all templates of Mb4Cas12a, as the structural reference for designing Mb4Cas12a mutations.

Liu et al. used an error-prone PCR to generate FnCas12a libraries and screen variants with high activity. They finally generated an eaFnCas12a with a 1.4-fold activity improvement. eaFnCas12a displayed similar specificity to wild-type FnCas12a, revealed by targeted sequencing of previously identified off-target sites. We previously measured FnCas12a specificity with the GFP-activation assay (PMID: 36763662), offering an opportunity to compare FnCas12a and Mb4Cas12a specificity side-by-side. FnCas12a substantially tolerates dinucleotide mismatches at spacer positions 3-24, while Mb4Cas12a is very sensitive to the dinucleotide mismatches at spacer positions 1-18 (Figure 5A). In addition, Mb4Cas12a is also sensitive to the single mismatches at number of spacer sites. Mb4Cas12a-F370 improved sensitivity to the single mismatches at spacer positions 9 and 11-15. From these data, we can conclude that Mb4Cas12a-F370 is more specific than eaFnCas12a.

> 5) Results - Analysis of Mb4Cas12a specificity: The authors state: "The results showed that these mutations had a small influence on Mb4Cas12a activity (Figure 5C-D)". This statement seems at odds with the actual results seen with the mutated Mb4Cas12a (Scale difference in Fig5A between Mb4Cas12a and its mutated version).

Reply: Thank you for the comments. We have cautiously rephrased this result. We state "The results showed that these mutations decreased editing activity".

> 6) Can the authors speculate on why Mb4Cas12a is active at temperatures as low as 10°C when compared to other Cas12a that have been shown to be active around 30°C? The source organism?

Reply: We can only speculate on the activity of cas9 from an evolutionary perspective. The source organism of Mb4Cas12a is Moraxella bovis CCUG 2133, separated from cattle with pinkeye. In addition to cattle, Moraxella bovis CCUG 2133 may live in many different environments, which requires Mb4Cas12a to function across a wide range of temperatures. Gasiunas et al. (PMID: 33139742) investigated a panel of 40 Cas9 enzymes and observed their functionality in temperatures ranging from 10°C to 68°C. For instance, SsaCas9, derived from Streptococcus salivarius JIM8777, retained 95% of its cleavage activity at 10°C. These findings suggest that some Cas nucleases can indeed function effectively at very low temperatures.

> 7) Overall, the authors need to improve the writing of the manuscript by elaborating further on their data, developing their ideas to a greater extent, and working on the transitions between sentences to improve fluidity. Understanding the logic and the conclusions of this paper will be difficult for those not already well-steeped in prior Cas12a literature.

Reply: We express our sincere gratitude to the reviewer for providing constructive comments on our manuscript. In response to the reviewer's suggestions, we have carefully revised and polished the manuscript to enhance its clarity and readability.

> Minor Points

> 1) Introduction: "CRISPR-Cas12a is particularly interesting because this system only contains two components: a Cas12a nuclease and a CRISPR RNA (crRNA)" … compared to what?

Reply: Thank you for your comments. We added more background information and stated "These distinctive features collectively position Cas12a as an excellent choice for genome editing applications".

> 2) Results - Efficient genome editing with Mb4Cas12a: the authors start this paragraph by mentioning that the Mb4Cas12a locus encodes for Cas4, Cas1, and Cas2. Is there a reason why the authors address this? There is no further development of this information in the results section and there is no added value to Fig4A.

Reply: Thank you for your comments. Following the reviewer's suggestions, we deleted it in the revised manuscript.

> 3) Results - Efficient genome editing with Mb4Cas12a: The authors pursue this by comparing Mb4Cas12a and LbCas12a activity at 13 TTTV PAM loci (Fig4D). Statistics are missing in (Fig4D).

Reply: Thank you for your suggestions. Following the reviewer's suggestions, we added the statistics in Figure 4D.

> 4) Results - Analysis of Mb4Cas12a specificity: "That Mb4Cas12a was sensitive to the dinucleotide mismatches (Fig. S5B). In sum, these data demonstrated that Mb4Cas12a did not tolerate dinucleotide mismatches". Redundant statements.

Reply: Thank you for your suggestions. In the revised manuscript, we deleted the "In sum, these data demonstrated that Mb4Cas12a did not tolerate dinucleotide mismatches".

> 5) Results - Analysis of Mb4Cas12a specificity: "These data indicated that Mb4Cas12a and F370A were very precise enzymes for genome editing". I would tone down "very precise" as both Mb4Cas12a and its mutant form F370A still showed off target effects (6 out of 27).

Reply: We are sorry for the vague statement. In the revised manuscript, "very" was deleted.

1.	Hu Z, Zhang C, Wang D, Gao S, Ong SG, Wang Y, et al. A Highly Sensitive GFP Activation Assay for Detection of DNA Cleavage in Cells. Front Cell Dev Biol. 2021;9:771248. Epub 2021/12/07. doi: 10.3389/fcell.2021.771248. PubMed PMID: 34869366; PubMed Central PMCID: PMCPMC8636026.

2.	Crooks GE, Hon G, Chandonia JM, Brenner SE. WebLogo: a sequence logo generator. Genome Res. 2004;14(6):1188-90. Epub 2004/06/03. doi: 10.1101/gr.849004. PubMed PMID: 15173120; PubMed Central PMCID: PMCPMC419797.

3.	Leenay RT, Maksimchuk KR, Slotkowski RA, Agrawal RN, Gomaa AA, Briner AE, et al. Identifying and Visualizing Functional PAM Diversity across CRISPR-Cas Systems. Mol Cell. 2016;62(1):137-47. Epub 2016/04/05. doi: 10.1016/j.molcel.2016.02.031. PubMed PMID: 27041224; PubMed Central PMCID: PMCPMC4826307.
