# Supplementary material for: Engineering of a high-fidelity Cas12a nuclease variant capable of allele-specific editing
Source: PLoS Biol. 2024 Jun 12;22(6):e3002680. doi: 10.1371/journal.pbio.3002680 (PMC11168656; doi:10.1371/journal.pbio.3002680)
Supplement: Attachment — Submitted filename: Response to Reviewers.docx [file pbio.3002680.s022.docx]

**Discovery and engineering of novel Cas12a nucleases for precision genome editing**

We express our sincere gratitude to the reviewers for their positive feedback and valuable suggestions to enhance our manuscript's quality. A copy of the comments (highlighted in grey) and a detailed point-by-point response can be found below. All changes and additions to the manuscript are marked for ease of review.

**REPLY TO REVIEWER #1:**

> Comments to the Author

> The authors have fully addressed my previous concerns.

Reply: Thank you for your positive comments on our manuscript.

**REPLY TO REVIEWER #2:**

> Comments to the Author

> The revised manuscript shows additional off-targets for POLQ (11 reads), which were absent in the original manuscript. The authors claim that the engineered Mb4Cas12a-F370A is more specific than its wild-type counterpart, but this is contradicted by the GUIDE-seq data presented in Figure 6. In fact, Mb4Cas12a-F370A seems far less specific than Mb4Cas12a based on the (proportionally) higher number of reads for POLQ off-targets 1, 2 and 3. Interestingly, these off-targets contain up to three mismatches but are still efficiently cleaved by Mb4Cas12a-F370A. The manuscript states: "LbCas12a generated 28 off-targets across six tested loci. In contrast, Mb4Cas12a generated six off-targets across six tested loci, while F370A generated seven off-targets across six tested loci.". However, no explanation is provided for this observation.

Reply: Thank you for your comments. In response to Reviewer #1's query from the initial review round, we reanalyzed the off-target effects, revealing an omission of off-targets for the POLQ locus (11 reads). Consequently, we have included this information in the revised manuscript. To ensure a fair comparison of off-target effects in the revised manuscript, we uniformly analyzed a comparable number of reads across all GUIDE-seq data.

Furthermore, to comprehensively assess the specificity of Mb4Cas12a and its F370A variant, we conducted GUIDE-seq analysis for additional sites. The results revealed that LbCas12a generated 59 off-targets across ten tested loci. In contrast, Mb4Cas12a generated 24 off-targets across the same ten tested loci, while F370A generated 19 off-targets across the identical set of loci. These findings demonstrate that Mb4Cas12a and its F370A variant exhibit superior specificity compared to the canonical LbCas12a.

The majority of off-targets were produced at the DNMT1-sg3 and POLQ sites. When targeting the DNMT1-sg3 site, Mb4Cas12a induced 18 off-target sites, while F370A produced 12 off-target sites (Fig. 6A). Mb4Cas12a demonstrated higher specificity than F370A at seven off-target sites, whereas F370A exhibited greater specificity than Mb4Cas12a at 18 off-target sites, based on reads number. Interestingly, we observed a bias in the off-targeting behaviour of Mb4Cas12a and its F370A variant when targeting the POLQ locus. For example, Mb4Cas12a exhibited higher specificity than F370A at the off1 site, whereas F370A displayed greater specificity than Mb4Cas12a at the off4 site (Fig. S10A). Similarly, at the off2 and off7 sites, which differed by only one nucleotide in the PAM region, Mb4Cas12a showed greater specificity than F370A at the off2 site, while F370A demonstrated higher specificity than Mb4Cas12a at the off7 site. These observations suggest potential variations in the off-targeting specificity of different Mb4Cas12a variants. It would be intriguing to investigate the specificity of other Mb4Cas12a variants further using GUIDE-seq in future studies.

**Fig. 6.** Test of Mb4Cas12a and F370A specificity with GUIDE-seq.

**Fig. S10.** Test of Mb4Cas12a and F370A specificity with GUIDE-seq.

**REPLY TO REVIEWER #3:**

> Comments to the Author

> The authors have sufficiently responded to prior comments, and this manuscript will be a nice addition to the field.

Reply: Thank you for your positive comments on our manuscript.
