# Supplementary material for: Engineering of a high-fidelity Cas12a nuclease variant capable of allele-specific editing
Source: PLoS Biol. 2024 Jun 12;22(6):e3002680. doi: 10.1371/journal.pbio.3002680 (PMC11168656; doi:10.1371/journal.pbio.3002680)
Supplement: Attachment — Submitted filename: Response to Editor.docx [file pbio.3002680.s023.docx]

**Engineering of a high-fidelity Cas12a nuclease variant capable of allele-specific editing**

We express our sincere gratitude to the editor. A copy of the comments (highlighted in grey) and a detailed point-by-point response can be found below. All changes and additions to the manuscript are marked for ease of review.

**REPLY TO EDITOR:**

Thank you for your patience while we considered your revised manuscript "Discovery and engineering of novel Cas12a nucleases for precision genome editing" for publication as a Research Article at PLOS Biology. This revised version of your manuscript has been evaluated by the PLOS Biology editors and the Academic Editor.

Based on our Academic Editor's assessment of your revision, I am pleased to say that we are likely to accept this manuscript for publication, provided you satisfactorily address the following data and other policy-related requests that I have provided below:

> (A) We would like to suggest the following modification to the title: “Engineering of a high-fidelity Cas12a nuclease variant capable of allele-specific editing”

Reply: According to the editor's suggestion, we have changed the title of the article to “Engineering of a high-fidelity Cas12a nuclease variant capable of allele-specific editing”.

> (B) In your conflict of interest statement in the online submission form, please provide additional details about the specific authors who have applied for the patent and any patent numbers which have been provided.

Reply: According to the editor's suggestion, we have provided additional details about the specific authors who have applied for the patent and the patent numbers which have been provided.

> (C) You may be aware of the PLOS Data Policy, which requires that all data be made available without restriction: <http://journals.plos.org/plosbiology/s/data-availability.> For more information, please also see this editorial: <http://dx.doi.org/10.1371/journal.pbio.1001797>. Thank you for already providing the raw data underlying the figures in the Table S5 file. However, we note that the raw data for Figure 5B is currently missing in the supplementary file?

Reply: According to the editor's suggestion, we have added the raw data for Figure 5B into the Table_S5 file.

> (D) Thank you for already providing the raw sequencing data in the Table S6 file. However, I note that the raw sequencing files for the GUIDE-seq data (Figure 6, S11), deep sequencing (Figure 2) and Sanger sequencing (Figure 3C) appears to be missing from this file. Given the potential size of the sequencing files, I would be grateful if you could please deposit this data in the GEO data repository. Please ensure that the data is made publicly available and provide the accession number in the Data Availability Statement in the online submission form.

Reply: According to the editor's suggestion, the raw data for the GUIDE-seq Data (Figure 6，S10) have been submitted to the NCBI Sequence Read Archive (SRA PRJNA: 1106073). The raw data for Sanger sequencing (Figure 3C) were packaged in the compressed supplemental file S2_Data in the revised manuscript. The raw data named “PAM” in the Table S6 file were the deep sequencing for Figure 2, then we changed the name “PAM” to “Figure 2B-2C”. All of the information on raw sequencing data is listed in Table_S6.

> (E) Please also ensure that each of the relevant figure legends in your manuscript include information on *WHERE THE UNDERLYING DATA CAN BE FOUND*, and ensure your supplemental data file/s has a legend.

Reply: According to the requirements, we have indicated the information on *WHERE THE UNDERLYING DATA CAN BE FOUND* in each of the relevant figure legends, and ensured our supplemental data file/s has a legend.

> (F) Please ensure that your Data Statement in the submission system accurately describes where your data can be found and is in final format, as it will be published as written there.

Reply: We accurately described in the data declaration where our data can be found.

> (G) Per journal policy, if you have generated any custom code during the curse of this investigation, please make it available without restrictions upon publication. Please ensure that the code is sufficiently well documented and reusable, and that your Data Statement in the Editorial Manager submission system accurately describes where your code can be found.

> Please note that we cannot accept sole deposition of code in GitHub, as this could be changed after publication. However, you can archive this version of your publicly available GitHub code to Zenodo. Once you do this, it will generate a DOI number, which you will need to provide in the Data Accessibility Statement (you are welcome to also provide the GitHub access information). See the process for doing this here: <https://docs.github.com/en/repositories/archiving-a-github-repository/referencing-and-citing-content>

> Reply: We have not generated any custom code during the curse of this investigation. Crispresso 2.0.31 was used for deep sequencing analysis, and guide-seq v1.0.2 was used for GUIDE-seq analysis. WebLogo 3.7.12 and a PAM wheel chart were used for PAM analysis.

> (H) We require the original, uncropped and minimally adjusted images supporting all blot and gel results reported in the following Figures: Figure 3A-B, 4B, S4A, S7B. We will require these files before a manuscript can be accepted so please prepare and upload them now. Please carefully read our guidelines for how to prepare and upload this data: <https://journals.plos.org/plosbiology/s/figures#loc-blot-and-gel-reporting-requirements>

Reply: According to the requirements, we have added the raw images underlying data about all blot and gel results, which showed in supplemental file S1_Data in the revised manuscript.

> (I) Please note that per journal policy, the specific species where the Cas12a nuclease was obtained from should be clearly stated in the abstract of your manuscript.

Reply: According to the per journal policy, we have stated the specific species where the Cas12a nuclease was obtained in the abstract of our revised manuscript.
